# 2-(4-Fluoro­phen­yl)quinoxaline

**DOI:** 10.1107/S1600536812017771

**Published:** 2012-05-19

**Authors:** Cui-Ping Wang, Saiyong Ma, Jiang-Long Yu, Jing-Bo Yan, Zhi-Qiang Zhang

**Affiliations:** a School of Chemical Engineering, University of Science and Technology Liaoning, Anshan 114051, People’s Republic of China

## Abstract

In the title compound, C_14_H_9_FN_2_, the dihedral angle between the benzene ring and the quinoxaline ring system is 22.2 (3)°. Any aromatic π–π stacking in the crystal must be very weak, with a minimum centroid–centroid separation of 3.995 (2) Å.

## Related literature

For background to the applications of quinoxaline derivatives, see: Lindsley *et al.* (2005 ▶); Dailey *et al.* (2001 ▶).
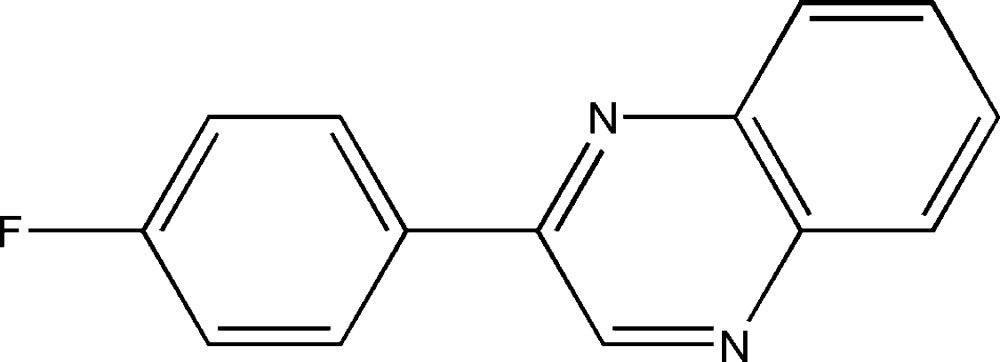


## Experimental

### 

#### Crystal data

C_14_H_9_FN_2_
*M* *_r_* = 224.23
Monoclinic, 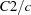

*a* = 24.249 (13) Å
*b* = 3.7925 (19) Å
*c* = 22.609 (13) Å
β = 91.866 (9)°
*V* = 2078.2 (19) Å^3^
*Z* = 8
Mo *K*α radiation
μ = 0.10 mm^−1^
*T* = 113 K
0.20 × 0.18 × 0.10 mm

#### Data collection

Rigaku Saturn724 CCD diffractometer
Absorption correction: multi-scan (*CrystalClear*; Rigaku, 2008 ▶) *T*_min_ = 0.981, *T*_max_ = 0.990
9711 measured reflections
2454 independent reflections
1797 reflections with *I* > 2σ(*I*)
*R*_int_ = 0.032

#### Refinement

*R*[*F* ^2^ > 2σ(*F* ^2^)] = 0.036
*wR*(*F* ^2^) = 0.109
*S* = 1.01
2454 reflections
154 parameters
H-atom parameters constrained
Δρ_max_ = 0.25 e Å^−3^
Δρ_min_ = −0.22 e Å^−3^

### 

Data collection: *CrystalClear* (Rigaku, 2008 ▶); cell refinement: *CrystalClear*; data reduction: *CrystalClear*; program(s) used to solve structure: *SHELXS97* (Sheldrick, 2008 ▶); program(s) used to refine structure: *SHELXL97* (Sheldrick, 2008 ▶); molecular graphics: *SHELXTL* (Sheldrick, 2008 ▶); software used to prepare material for publication: *SHELXTL*.

## Supplementary Material

Crystal structure: contains datablock(s) global, I. DOI: 10.1107/S1600536812017771/hb6725sup1.cif

Structure factors: contains datablock(s) I. DOI: 10.1107/S1600536812017771/hb6725Isup2.hkl

Supplementary material file. DOI: 10.1107/S1600536812017771/hb6725Isup3.cml

Additional supplementary materials:  crystallographic information; 3D view; checkCIF report

## Figures and Tables

**Fig. 1.**
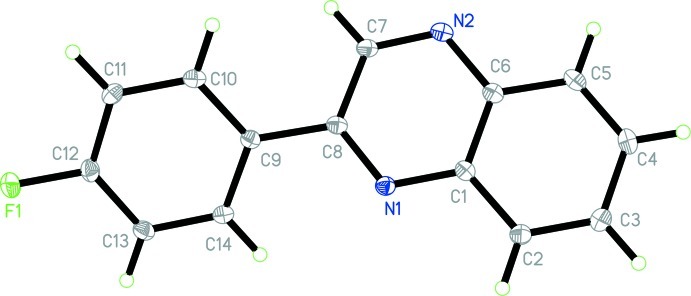
The molecular structure of the title compound, showing 30% probability displacement ellipsoids for the non-hydrogen atoms.

## supplementary crystallographic information

### Comment

Quinoxaline and its derivatives are an important class of nitrogen-containing heterocycles displaying both biologial activities (Lindsley *et al.*, 2005) and technological applications (Dailey *et al.*, 2001). Here, we report the synthesis and crystal structure of the title compound (Fig. 1).

In the title compound, C_14_H_9_FN_2_, the dihedral angle between the benzene ring and the quinoxaline ring is 22.2 (3)°.

### Experimental

A solution of benzene-1,2-diamine (1.5 mmol) and 2-(4-fluorophenyl)-2-oxoacetaldehyde monohydrate (1.5 mmol) in EtOH (10 ml) was stirred at room temperature for 0.5 h. After completion of the reaction (monitored by TLC or HPLC), the precipitated solid was collected by filtration and dried to afford the pure product. Or after completion of the reaction, water was added to the reaction mixture and filtered to afford the product. When necessary, the product was recrystallized from ethanol/water. Colourless prisms were grown by slow evaporation of a solution in chloroform/ethanol (1:1).

### Refinement

H atoms were placed in calculated positions (C—H = 0.95 Å) and refined as riding with *U*_iso_(H) = 1.2*U*_eq_(C).

### Crystal data

**Table:**

C_14_H_9_FN_2_	*F*(000) = 928
*M**_r_* = 224.23	*D*_x_ = 1.433 Mg m^−^^3^
Monoclinic, *C*2/*c*	Mo *K*α radiation, λ = 0.71073 Å
*a* = 24.249 (13) Å	Cell parameters from 3509 reflections
*b* = 3.7925 (19) Å	θ = 1.7–27.9°
*c* = 22.609 (13) Å	µ = 0.10 mm^−^^1^
β = 91.866 (9)°	*T* = 113 K
*V* = 2078.2 (19) Å^3^	Prism, colorless
*Z* = 8	0.20 × 0.18 × 0.10 mm

### Data collection

**Table:**

Rigaku Saturn724 CCD diffractometer	2454 independent reflections
Radiation source: rotating anode	1797 reflections with *I* > 2σ(*I*)
Multilayer monochromator	*R*_int_ = 0.032
Detector resolution: 14.22 pixels mm^-1^	θ_max_ = 27.9°, θ_min_ = 1.7°
ω and φ scans	*h* = −31→31
Absorption correction: multi-scan (*CrystalClear*; Rigaku, 2008)	*k* = −4→4
*T*_min_ = 0.981, *T*_max_ = 0.990	*l* = −29→29
9711 measured reflections	

### Refinement

**Table:**

Refinement on *F*^2^	Primary atom site location: structure-invariant direct methods
Least-squares matrix: full	Secondary atom site location: difference Fourier map
*R*[*F*^2^ > 2σ(*F*^2^)] = 0.036	Hydrogen site location: inferred from neighbouring sites
*wR*(*F*^2^) = 0.109	H-atom parameters constrained
*S* = 1.01	*w* = 1/[σ^2^(*F*_o_^2^) + (0.0692*P*)^2^] where *P* = (*F*_o_^2^ + 2*F*_c_^2^)/3
2454 reflections	(Δ/σ)_max_ = 0.001
154 parameters	Δρ_max_ = 0.25 e Å^−^^3^
0 restraints	Δρ_min_ = −0.22 e Å^−^^3^

### Special details

**Table:**

Geometry. All e.s.d.'s (except the e.s.d. in the dihedral angle between two l.s. planes) are estimated using the full covariance matrix. The cell e.s.d.'s are taken into account individually in the estimation of e.s.d.'s in distances, angles and torsion angles; correlations between e.s.d.'s in cell parameters are only used when they are defined by crystal symmetry. An approximate (isotropic) treatment of cell e.s.d.'s is used for estimating e.s.d.'s involving l.s. planes.
Refinement. Refinement of *F*^2^ against ALL reflections. The weighted *R*-factor *wR* and goodness of fit *S* are based on *F*^2^, conventional *R*-factors *R* are based on *F*, with *F* set to zero for negative *F*^2^. The threshold expression of *F*^2^ > σ(*F*^2^) is used only for calculating *R*-factors(gt) *etc*. and is not relevant to the choice of reflections for refinement. *R*-factors based on *F*^2^ are statistically about twice as large as those based on *F*, and *R*- factors based on ALL data will be even larger.

### Fractional atomic coordinates and isotropic or equivalent isotropic displacement parameters (Å^2^)

**Table:**

	*x*	*y*	*z*	*U*_iso_*/*U*_eq_	
F1	0.46580 (3)	0.67692 (19)	1.18737 (3)	0.0321 (2)	
N1	0.39311 (3)	0.1470 (2)	0.93164 (4)	0.0184 (2)	
N2	0.28374 (3)	−0.1127 (2)	0.93193 (4)	0.0213 (2)	
C1	0.36818 (4)	0.0119 (3)	0.88147 (4)	0.0182 (2)	
C2	0.39774 (4)	−0.0011 (3)	0.82863 (5)	0.0216 (3)	
H2	0.4347	0.0813	0.8283	0.026*	
C3	0.37306 (5)	−0.1322 (3)	0.77801 (5)	0.0238 (3)	
H3	0.3931	−0.1408	0.7426	0.029*	
C4	0.31805 (5)	−0.2552 (3)	0.77785 (5)	0.0246 (3)	
H4	0.3013	−0.3434	0.7423	0.030*	
C5	0.28877 (4)	−0.2481 (3)	0.82842 (5)	0.0226 (3)	
H5	0.2519	−0.3332	0.8280	0.027*	
C6	0.31319 (4)	−0.1147 (3)	0.88123 (5)	0.0189 (2)	
C7	0.30886 (4)	0.0153 (3)	0.97928 (5)	0.0205 (2)	
H7	0.2895	0.0180	1.0152	0.025*	
C8	0.36398 (4)	0.1514 (3)	0.97994 (5)	0.0175 (2)	
C9	0.38994 (4)	0.2927 (3)	1.03511 (4)	0.0173 (2)	
C10	0.35866 (4)	0.4178 (3)	1.08148 (5)	0.0204 (3)	
H10	0.3195	0.4132	1.0779	0.025*	
C11	0.38416 (5)	0.5485 (3)	1.13269 (5)	0.0217 (3)	
H11	0.3630	0.6352	1.1642	0.026*	
C12	0.44101 (5)	0.5499 (3)	1.13683 (5)	0.0217 (3)	
C13	0.47352 (4)	0.4309 (3)	1.09221 (5)	0.0220 (3)	
H13	0.5126	0.4356	1.0963	0.026*	
C14	0.44741 (4)	0.3042 (3)	1.04120 (5)	0.0193 (2)	
H14	0.4690	0.2235	1.0096	0.023*	

### Atomic displacement parameters (Å^2^)

**Table:**

	*U*^11^	*U*^22^	*U*^33^	*U*^12^	*U*^13^	*U*^23^
F1	0.0308 (4)	0.0440 (5)	0.0213 (4)	−0.0068 (3)	−0.0033 (3)	−0.0063 (3)
N1	0.0176 (4)	0.0186 (5)	0.0190 (4)	−0.0001 (4)	0.0009 (3)	0.0012 (4)
N2	0.0164 (4)	0.0220 (5)	0.0256 (5)	−0.0004 (4)	0.0011 (4)	0.0017 (4)
C1	0.0184 (5)	0.0157 (5)	0.0204 (5)	0.0005 (4)	−0.0014 (4)	0.0015 (4)
C2	0.0208 (5)	0.0212 (6)	0.0228 (6)	−0.0016 (4)	0.0017 (4)	0.0009 (4)
C3	0.0282 (6)	0.0227 (6)	0.0206 (5)	0.0005 (5)	0.0023 (4)	0.0004 (5)
C4	0.0279 (6)	0.0225 (6)	0.0229 (6)	0.0010 (5)	−0.0066 (4)	−0.0017 (5)
C5	0.0183 (5)	0.0208 (6)	0.0284 (6)	0.0000 (4)	−0.0045 (5)	−0.0002 (5)
C6	0.0180 (5)	0.0152 (5)	0.0233 (5)	0.0018 (4)	−0.0011 (4)	0.0023 (4)
C7	0.0164 (5)	0.0225 (6)	0.0226 (5)	−0.0002 (4)	0.0021 (4)	0.0026 (4)
C8	0.0156 (5)	0.0161 (6)	0.0207 (5)	0.0020 (4)	0.0002 (4)	0.0031 (4)
C9	0.0177 (5)	0.0159 (5)	0.0182 (5)	−0.0010 (4)	0.0007 (4)	0.0034 (4)
C10	0.0163 (5)	0.0224 (6)	0.0227 (5)	0.0008 (4)	0.0022 (4)	0.0032 (4)
C11	0.0245 (5)	0.0228 (6)	0.0183 (5)	0.0011 (5)	0.0050 (4)	0.0013 (4)
C12	0.0260 (5)	0.0221 (6)	0.0169 (5)	−0.0035 (4)	−0.0024 (4)	0.0007 (4)
C13	0.0176 (5)	0.0247 (6)	0.0235 (5)	−0.0008 (4)	−0.0010 (4)	0.0018 (5)
C14	0.0176 (5)	0.0196 (6)	0.0208 (5)	0.0007 (4)	0.0027 (4)	0.0012 (4)

### Geometric parameters (Å, º)

**Table:**

F1—C12	1.3617 (13)	C5—H5	0.9500
N1—C8	1.3198 (14)	C7—C8	1.4325 (15)
N1—C1	1.3676 (14)	C7—H7	0.9500
N2—C7	1.3078 (15)	C8—C9	1.4792 (16)
N2—C6	1.3703 (14)	C9—C14	1.3966 (15)
C1—C2	1.4138 (16)	C9—C10	1.3969 (15)
C1—C6	1.4171 (16)	C10—C11	1.3864 (16)
C2—C3	1.3676 (16)	C10—H10	0.9500
C2—H2	0.9500	C11—C12	1.3787 (17)
C3—C4	1.4131 (17)	C11—H11	0.9500
C3—H3	0.9500	C12—C13	1.3764 (16)
C4—C5	1.3656 (16)	C13—C14	1.3835 (15)
C4—H4	0.9500	C13—H13	0.9500
C5—C6	1.4095 (16)	C14—H14	0.9500
			
C8—N1—C1	117.21 (9)	C8—C7—H7	118.3
C7—N2—C6	116.44 (10)	N1—C8—C7	120.74 (10)
N1—C1—C2	119.44 (10)	N1—C8—C9	118.54 (10)
N1—C1—C6	121.36 (10)	C7—C8—C9	120.71 (9)
C2—C1—C6	119.20 (10)	C14—C9—C10	118.67 (10)
C3—C2—C1	119.98 (11)	C14—C9—C8	119.37 (9)
C3—C2—H2	120.0	C10—C9—C8	121.96 (10)
C1—C2—H2	120.0	C11—C10—C9	120.66 (10)
C2—C3—C4	120.63 (10)	C11—C10—H10	119.7
C2—C3—H3	119.7	C9—C10—H10	119.7
C4—C3—H3	119.7	C12—C11—C10	118.41 (10)
C5—C4—C3	120.52 (10)	C12—C11—H11	120.8
C5—C4—H4	119.7	C10—C11—H11	120.8
C3—C4—H4	119.7	F1—C12—C13	118.89 (10)
C4—C5—C6	120.05 (10)	F1—C12—C11	118.13 (10)
C4—C5—H5	120.0	C13—C12—C11	122.98 (10)
C6—C5—H5	120.0	C12—C13—C14	117.86 (10)
N2—C6—C5	119.65 (10)	C12—C13—H13	121.1
N2—C6—C1	120.73 (10)	C14—C13—H13	121.1
C5—C6—C1	119.61 (10)	C13—C14—C9	121.40 (10)
N2—C7—C8	123.50 (10)	C13—C14—H14	119.3
N2—C7—H7	118.3	C9—C14—H14	119.3
			
C8—N1—C1—C2	−179.57 (9)	C1—N1—C8—C9	179.40 (9)
C8—N1—C1—C6	0.65 (15)	N2—C7—C8—N1	−1.44 (17)
N1—C1—C2—C3	−179.32 (10)	N2—C7—C8—C9	179.95 (10)
C6—C1—C2—C3	0.47 (16)	N1—C8—C9—C14	−21.66 (15)
C1—C2—C3—C4	0.10 (17)	C7—C8—C9—C14	156.99 (10)
C2—C3—C4—C5	−0.63 (17)	N1—C8—C9—C10	157.91 (10)
C3—C4—C5—C6	0.56 (17)	C7—C8—C9—C10	−23.44 (16)
C7—N2—C6—C5	−179.90 (10)	C14—C9—C10—C11	−0.46 (16)
C7—N2—C6—C1	0.90 (15)	C8—C9—C10—C11	179.97 (10)
C4—C5—C6—N2	−179.19 (10)	C9—C10—C11—C12	−0.40 (16)
C4—C5—C6—C1	0.02 (16)	C10—C11—C12—F1	−179.66 (10)
N1—C1—C6—N2	−1.55 (16)	C10—C11—C12—C13	0.72 (17)
C2—C1—C6—N2	178.67 (9)	F1—C12—C13—C14	−179.76 (9)
N1—C1—C6—C5	179.25 (9)	C11—C12—C13—C14	−0.15 (18)
C2—C1—C6—C5	−0.53 (15)	C12—C13—C14—C9	−0.76 (16)
C6—N2—C7—C8	0.53 (16)	C10—C9—C14—C13	1.06 (16)
C1—N1—C8—C7	0.76 (15)	C8—C9—C14—C13	−179.36 (9)

## References

[bb1] Dailey, S., Feast, J. W., Peace, R. J., Sage, I. C., Till, S. & Wood, E. L. (2001). *J. Mater. Chem.* **11**, 2238–2243.

[bb2] Lindsley, C. W., Zhao, Z., Leister, W. H., Robinson, R. G., Barnett, S. F., Defeo-Jones, D., Jones, R. E., Hartman, G. D., Hu, J. R., Huber, H. E. & Duggan, M. E. (2005). *Bioorg. Med. Chem. Lett.* **15**, 761–764. 10.1016/j.bmcl.2004.11.011 15664853

[bb3] Rigaku (2008). *CrystalClear* Rigaku Corporation, Tokyo, Japan.

[bb4] Sheldrick, G. M. (2008). *Acta Cryst.* A**64**, 112–122. 10.1107/S0108767307043930 18156677

